# Recognizing Significant Components of Electrical Waveforms of Actuators Operated by Vehicle Controllers

**DOI:** 10.3390/s22207945

**Published:** 2022-10-18

**Authors:** Krzysztof Więcławski, Maja Antkowiak, Tomasz Figlus

**Affiliations:** 1 Faculty of Automotive and Construction Machinery Engineering, Warsaw University of Technology, 05-524 Warsaw, Poland; 2 Faculty of Transport and Aviation Engineering, Silesian University of Technology, 40-019 Katowice, Poland

**Keywords:** fuel injector, fuel system, combustion engine, electrical properties, mechanical, diagnosing

## Abstract

This work discusses the proposition of the identification of electrical waveforms resulting from the executive systems’ operation, controlled by the vehicle electronic modules. This proposition results from the fact that the electric current powering the actuator has two functions: to supply and to control. Observation of such waveforms enables the ongoing control as well as the diagnostics of the state of the executive elements. This work focused on the fundamentals of the method implementation in the vehicle controller. The algorithm for detecting the model values of the waveform has been developed and described, allowing for an efficient control of the system. The algorithm, after being used in the memory of the control module and having the measuring subassemblies (gauging the voltage and electric current) added, will enable the automatic detection of the essential values. The developed code, after the optimization, can support the control performed by the ECU, which is damage-orientated. The paper presents examples of the operation of a computational program developed on the basis of the adopted algorithm. Tests were performed on an electromagnetic valve—fuel injector—of a spark-ignition engine for different cases of its operation. The effectiveness of the program was demonstrated when detecting changes occurring in the current signals of fuel injectors corresponding to different engine speeds (time of 4 and 8 ms) and different loads (pressure of 0.2 and 0.4 MPa).

## 1. Introduction

Control of the executive elements is based on the defined range of applied parameters matching each other, which determines the adequate functioning of the system. In the case of the combustion engine, the actuator is the fuel injector, and if properly controlled, ensures the economy of the fuel consumption, minimization of the amount of toxic substances resulting from the engine operation, and the optimal engine power. Controlling the digital actuator makes it possible to achieve a precisely defined goal, with adequately fast variations that correspond to changes in the parameters of the combustion process.

Ensuring proper operating conditions for internal combustion engines requires further development of the already developed and known tools as well as undertaking new research to advance knowledge of the information contained in the current signals generated by injectors. These activities influence the development of tools related to the ongoing correction of injectors as well as the assessment of their technical condition during normal operation.

### 1.1. Analysis of the State-of-the-Art in Testing Fuel Injectors and Engines

Hu et al. [1] described the influence of the structural parameters of the electronic fuel injector on its dynamic response (delay in the opening and delay in the closing). The model of the electronic fuel injector was referred to, which was integrated with the optimal model. The significance and impact of the main structural parameters on the actuator’s dynamic response was tested. It has been determined that the geometry of the needle and of the nozzle channel affects the delay in the actuator’s operation. What causes the above is also the electric current phenomena such as the increase in the current intensity and in the resultant magnetic flux around the injector core [2]. The work of Sun et al. [3] discussed the developed algorithm for recognition of the characteristics of the opening and shutting off the fuel flow; the method of auto-adaptation of the flow characteristics was proposed as well as the method for compensation in the width of the injection control impulse on the basis of the negative-loop signal of the voltage controlling the injector. The characteristics of the fuel flow through the nozzle is the result of the needle dynamics [4,5]. The function of the process control and the monitoring of the values fed allows for the diagnostics of the process, which is why measuring the electric current flowing through the injector coil is a superb tool for this purpose [6,7,8], due to which the detection of failures in the fuel system is possible. Analyses of the variable control of fuel flow in a high-pressure injector and its effect on atomization were analyzed in [9]. Examples of research on electromagnetic actuators and the usefulness of analyzing electrical signals in the process of diagnosing cars are presented in [10].

The control parameters of internal combustion engine power systems have a significant impact on their operation. Appropriate adjustment of the parameters of the gasoline and gas supply control algorithm affects the emission of toxic components of the exhaust gas [11,12,13], and also affects the dynamic parameters of the car [14,15,16]. Research on the selection of injection parameters and optimization of the EGR of an engine fueled with a biofuel admixture is presented in [17]. In [18], the authors presented research on the development of an electronic control unit (ECU) for a four-cylinder petrol engine, with the goal of improving engine performance. In this research, a model designed in Simulink was used. Interesting research work on the engine control system was presented in [19,20], where a new control system for the combustion process of high-speed engines was developed. Research on a model-based closed-loop injection flow rate controller for a piezoelectric fuel injector was presented in [21]. Simulation studies on flow and cavitation phenomena in a high-pressure control valve and the effect of the control valve structure parameters on the flow and cavitation characteristics were presented in [22,23]. Measurement studies and theoretical analyses on the displacement characteristics of the moving components of injectors were carried out by the authors of [24]. Research in the field of diagnosing internal combustion engine components and the impact of their condition on engine operation can also be found in [25,26,27,28,29,30,31]. These studies demonstrate the effectiveness of diagnosis conducted on the basis of the analysis of recorded vibration [26,27,28,29,30] and acoustic signals [29,31] as well as the use of advanced signal processing.

### 1.2. Basis for Conducting Research and Its Scope

The scientific work presented in Section 1.1 shows that research into internal combustion engines, injection systems, and the injectors themselves needs to be continued. One of the important directions of research on their development is the elaboration of systems for automatic diagnosing of the condition of actuator valves as components that have a significant impact on the operation of engines, fuel consumption, and emissions of toxic exhaust components. The authors’ previous research into analyzing current signals of internal combustion engines [2,8,32,33], especially fuel injectors, has led to a conclusion that in the considered case of injector research, there is an unambiguous lack of software of this type for analyzing the current values of injectors in an automatic manner during their operation on the engine. The development of these systems may contribute to the emergence/expansion of self-adjustment or adaptive systems relative to their momentary technical condition in the future. The authors are working on developing and implementing the research methods [2,8,32,33] established in the past. The direction of current work is primarily the development of software for its implementation in the ECU. Ongoing automatic monitoring is valuable for observing injector performance and its momentary technical condition. The development of new codes for analyzing current signals of internal combustion engines can be used to further develop control systems in the direction of condition diagnosis.

This paper presents a developed method for the automatic processing of the recorded current values of solenoid valves, which makes it possible to analyze their condition during normal operation. The first part of the study discusses the most important characteristic points of the current waveform of a fuel injector, useful in the process of its diagnosis and determination of its technical condition. The next section discusses in detail the theoretical assumptions necessary for the development of a system for the automatic determination of the current–voltage characteristics of an electromagnetic valve. Then, we present and discuss selected parts of the computational algorithm determining the characteristic features. In the later part of the study, on the basis of calculations carried out with the developed program, the sample results of tests on fuel injectors of a spark-ignition internal combustion engine are presented in graphs and tables.

## 2. Methodology

The following research methodology was adopted in this study:

1. Research goal
  To develop a program for the automatic calculation of the characteristics of the current signals of injectors.
2. Investigated object
  Fuel injector of the internal combustion engine.
3. Scope of research
  (a) Defining the basic components of the characteristics of electric current waveforms occurring during the operation of internal combustion engine fuel injectors as well as evaluating the possibility of their use in the analysis of the operation and technical condition. The research will be conducted on the basis of the literature analysis and the results of the authors’ research work presented in other publications.
  (b) Adoption of computational assumptions and the analysis of mathematical relationships required to develop the algorithm of the computational program for processing recorded electrical signals and determining the relevant information. On the basis of the analysis to be carried out, the dependencies will be selected, which enable the extraction of the characteristics of current signals, forming the basis for further scientific research.
  (c) Development of a computational program for determining and analyzing the required values of the recorded current signals of fuel injectors, along with a method for presenting the results. An assumption was made that the program should allow for clear implementation of the selected mathematical dependencies as well as the rapid calculation of current signals and the visualization of test results in the form of a graph and in a table.
  (d) Testing of the developed computational program on the basis of previously recorded current signals of fuel injectors as well as presentation of the sample test results. These tests will be conducted for injectors operating at different engine speeds and with different loads.
  (e) Discussion of the research conducted and the results obtained.

## 3. Characteristic Values of the Electric Current Waveform

**Figure 1:**
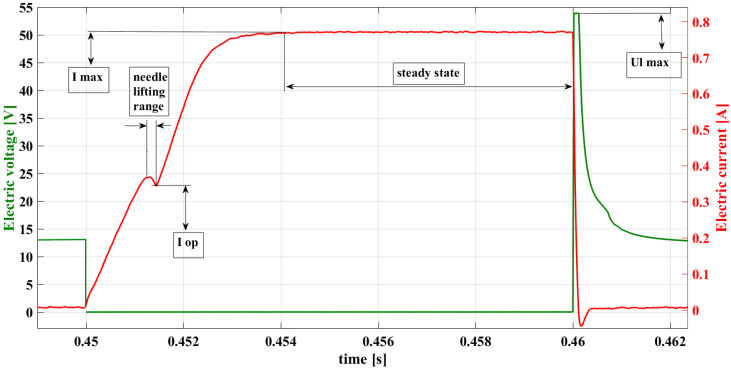
Characteristic points of a sample current waveform.

The verification method of the fuel injector operation and state, based on the observation of electric current waveforms, comes down to a comparison of the model waveform, resulting from the determined control parameters. However, the comparison of the whole current waveform is not required. Monitoring of the characteristic points in the current waveform, as shown in Figure 1, is sufficient. These points are as follows:

$I_{op}$—electric current intensity at the point of needle lifting [A];

$I_{max\;}$—value of electric current intensity in steady state (maximum) [A];

$U_{L,max}$—value of electric voltage at the maximum of induction peak [V];

$t_{I_{op}}$—value occurrence phase $I_{op}$ [s].

Parameter $I_{op}$ results from the level of fuel pressure before the injector and from the electrical system efficiency (i.e., the power supply and injector coil). The level of value $I_{max}$ is connected to the state of the electrical system (with the resistance). A drop in the value of $U_{L,max}$ (i.e., of the inductance jump after shutting off the control impulse) indicates the occurrence of a short circuit in the electrical system. What is meant here is a short circuit of a certain (low) degree, that is, at the initial phase of failure. In the case of changing time phases such as the extension of time $t_{I_{op}}$ may result from the electrical failure. The theoretical basis and examples of research results in this area are widely discussed by the authors in [33].

The purpose of this work was to propose the automatic detection of essential values in the voltage–current waveform, which would enable this method’s implementation in the control system (ECU) memory. The injector control is accomplished by means of the controller on the basis of the memory areas in the digitally managed microcontrollers, hence the remainder of the paper presents the theoretical basis necessary for the development of the computational algorithm that forms the basis of the computational program.

## 4. Assumptions of the Computational Algorithm

An analytical search for the values mentioned in the previous section was based on the determination of the extrema of the function of the electric current and voltage in the ranges of their occurrence. According to the Weierstrass theorem [34], if function f: 〈a, b〉→R, is a continuous function, there exists the smallest (m) and the greatest (M) values of function f in the closed interval <a, b>. Hence, the minimum must exist $\left(x_{1}\;\epsilon \langle a,\;b\rangle \right)$ (1) as well as the function maximum $\left(x_{2}\;\epsilon \langle a,\;b\rangle \right)$ (2)

(1) $$f\left(x_{1}\right)=m\;$$

(2) $$f\left(x_{2}\right)=M$$

According to the Fermat theorem [34], the derivative of the function at its extremum is equal to zero. Based on this theorem, the potentially extreme points can be determined. The zero derivative of a function does not indicate that there is an extremum at a given point, it is only a necessary condition for its occurrence. If function f:(a, b) → R, it is differentiable at a point $x_{0}$ *ϵ* (*a*, *b*), and

(3) $$f\left(x_{0}\right)=max\left\{f\left(x\right):x\;\epsilon \left(a,\;b\right)\right\}$$

or

(4) $$f\left(x_{0}\right)=min\left\{f\left(x\right):x\;\epsilon \left(a,\;b\right)\right\}$$

then the zero derivative of function $f\prime \left(x_{0}\right)=0$ is a necessary condition for the occurrence of an extremum, and a sufficient condition is the sign of this derivative $f\prime \prime \left(x_{0}\right)>0$. Sequentially applying the Weierstrass and Fermat [34] theorems allows for the determination of the potentially extreme points. The Fermat’s theorem invoked here specifies that if function *y = f(x)* is given in a coherent interval and has at some point inside this interval *(x = c)* the highest or the lowest value, it is such that:

(5) f(c)>f(x)

or

(6) f(c)<f(x)

and has a finite derivative at a point, the derivative at that point equals zero:

(7) $$f^{\prime }\left(c\right)=0$$

Due to the sufficient condition, it is possible to analytically verify and determine the function’s local minimum or maximum. Finding the greatest (or smallest) value of the function (8):

(8) $$u=f\left(x_{1},x_{2},\ldots ,x_{n}\right)\;$$

in a selected area requires locating all internal, potentially extreme points as well as computing the function’s value in this area and comparing it with the function’s value at the boundary points. The principles of locating the function’s minimum have been used to automatically find the value $I_{op}$ (Figure 1). Equation of the function describing the range of the needle lifting (9) [2]:

(9) $$I\left(t\right)=f_{press}*\varepsilon_{0}*e^{-\frac{R}{L_{2}}*t}+f_{p}$$

where

*L*—inductance [*H*];

*R*—resistance [Ω];

$\varepsilon_{0}$—electromotive force [*V*];

*t*—time [*s*];

$f_{press}$—coefficient dependent on injection pressure;

$f_{p}$—position coefficient;

*e*—the Euler number.

The change in the injector core inductance from $L_{1}$ to $L_{2}$ related to the needle shift takes place within this range. To facilitate the computations, parameter $L_{2}$ was adopted in the equation. The local minimum defines the state, when the derivative of a function is equal to zero.

(10) $$I^{\prime }\left(t\right)=f_{press}*\varepsilon_{0}*\left(-\frac{R}{L_{2}}\right)*e^{-\frac{R}{L_{2}}*t}$$

Derivative I(t) (10) tends to zero because the component $e^{-\frac{R}{L_{2}}*t}$ for t→∞ tends to zero. Applying the monotonicity condition of the function ensues that function I(t) is monotonically decreasing, therefore it does not have an extremum, and the lowest value the function takes at the end of the interval. The point sought for within the range of the needle lifting $I_{op}$ (Figure 1) for the values on the abscissa at point $t_{op}$:

(11) $$I\left(t_{op}\right)=I_{op}$$

Finding the current intensity value in the steady state $I_{max}$ (constant function) is based on the determination of the monotonicity of the function and the value of its derivative. The function is constant only when its derivative is equal to zero for the entire interval of the function (Figure 1).

(12) f′(x)=0 

The function determining the range of the current waveform from point $I_{op}$ to the end of the PWM (pulse-width modulation) control impulse (tending to the steady state) is defined by Equation (13) [2]:

(13) $$I\left(t\right)=f_{press}*\frac{\varepsilon_{0}}{R}\left(1-e^{\left(-\frac{R}{L_{2}}t\right)\;}\right)-f_{p}$$

The function derivative (13) is:

(14) $$I^{\prime }\left(t\right)=\frac{f_{press}*\varepsilon_{0}*R*e^{-\frac{R*t}{L_{2}}}}{L_{2}*R}$$

The derivative value (14) tends to zero and function (13) is increasing, thus the maximal value of the current intensity for the steady state is located within the function at the end of the PWM interval (Figure 1).

The maximal value of the inductive jump $U_{max}$ is determined by Equation (15):

(15) $$\left|U_{L}\right|=-L\frac{dI}{dt}$$

where $\frac{dI}{dt}$ is the velocity of changes in the current intensity over time.

A high derivative value in the intensity of the deteriorating current in a short timespan (release of high energy) in which the resistance tends to infinity (R →∞)  and the current intensity decreases to zero  (I→0) causes the inductive jump in the voltage $U_{L}$ to the value of approx. 56 V (at the source supply U=12 V). The voltage change starting from the point of the PWM impulse termination is represented by Equation (16):

(16) $$\left|U_{L}\right|=\varepsilon_{0}*e^{-\frac{R}{L_{2}}t}$$

The maximal value needs to be localized in this equation. Equation (16) is a decreasing function and tends to zero. In the discussed case, the voltage ($U_{L}$) decreases from the value of approx. 56 V to the level of supply (approx. 12 V). What results from the Fermat theorem [34] is that the function’s extremum is at the point where the function derivative is equal to zero (i.e., at the beginning of the range of the voltage drop (16)).

Based on the definitions given, the algorithm was developed to automatically seek the characteristic values of the current waveform, as described above: $I_{op}$ [A], $I_{max}$ [A] and $U_{L,max}$ [V] (Figure 1). The developed code will allow for a future implementation in the digital control modules.

## 5. The Algorithm and the Computational Program

Based on the adopted computational assumptions, an electrical signal processing algorithm and computational program were developed in the MATLAB environment.

**Figure 2:**
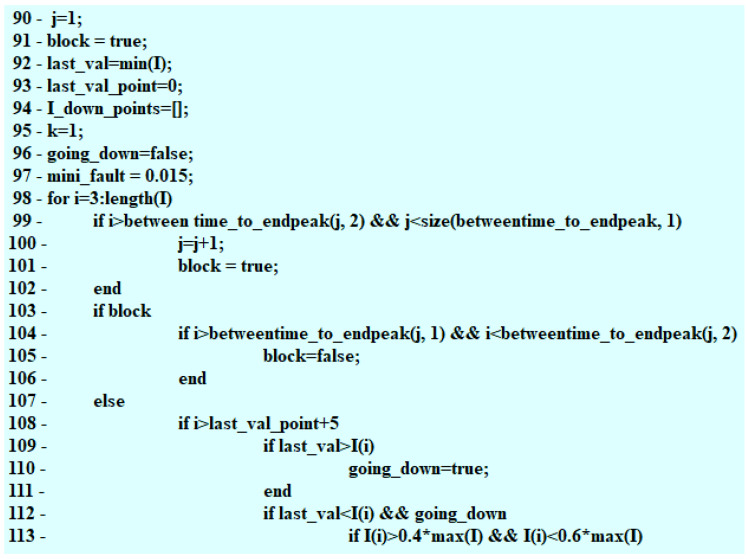
Observation of the changes in current intensity.

The construction of the algorithm started with defining the necessary variables, time intervals of the steady state duration, and the value of the current intensity occurring at that moment. The algorithm keeps “observing” the voltage waveform determining the moment, when the value of voltage drops to 0 V (Figure 2) and the point in time (s), when the voltage peak (V) takes place. The algorithm verifies whether the detected action took place after the drop in the voltage value to 0 V for every injection. Due to this, a PWM is determined and a maximal jump (inductive) after the PWM impulse is terminated. The code designed to find the maximal voltage value operates within a single loop. Subsequently, by means of comparing the samples, the moments of sudden voltage jumps are located and assigned to the previously implemented vector U_up_times. In the successive iterations, changes in the current waveform are detected, starting from finding the maximal value of the steady state I(t) (Figure 2).

**Figure 3:**
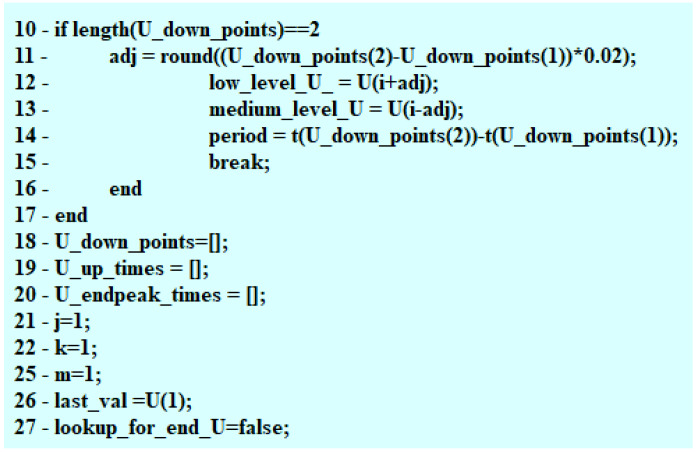
Fragments of code related to the maximal value of the electric voltage.

The maximal voltage value is a value detected in a single loop. Moving along the values of the vector U enables locating the spots where a sudden increase in voltage happened (Figure 3) with the values obtained recorded in the program memory. The change (decrease) in the maximal value (inductive jump) informs the user about a short in the circuit.

**Figure 4:**
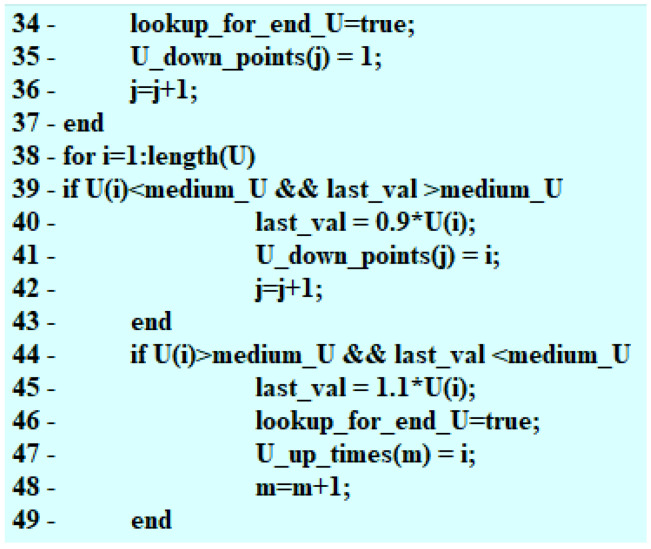
Selection of the search range.

Operation of the algorithm makes it possible to define the point of the start of the fuel flow—the range of the needle lifting. The foundation of the algorithm are invariably the samples taken on the basis of the voltage waveform. The algorithm, moving along the voltage waveform, compares the voltage values to the mean value. The numbers of samples including the voltage changes from 12 V to 0 V are registered because the points that are sought are only to be found within the range of operation of the control PWM pulse (Figure 4).

**Figure 5:**
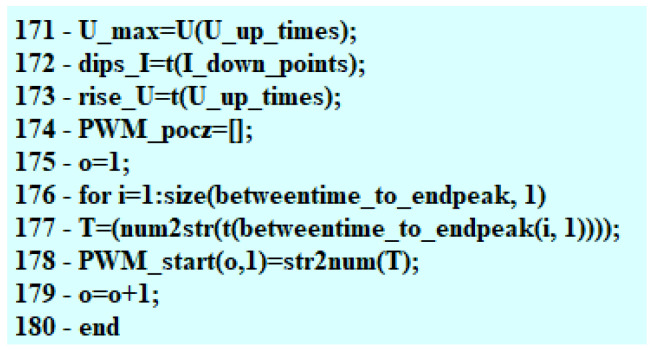
Searching for the sudden voltage jumps.

Successive samples are compared in the code, due to which the moments of sudden voltage jumps are detected and assigned to the previously implemented vector U_up_times (Figure 5).

**Figure 6:**
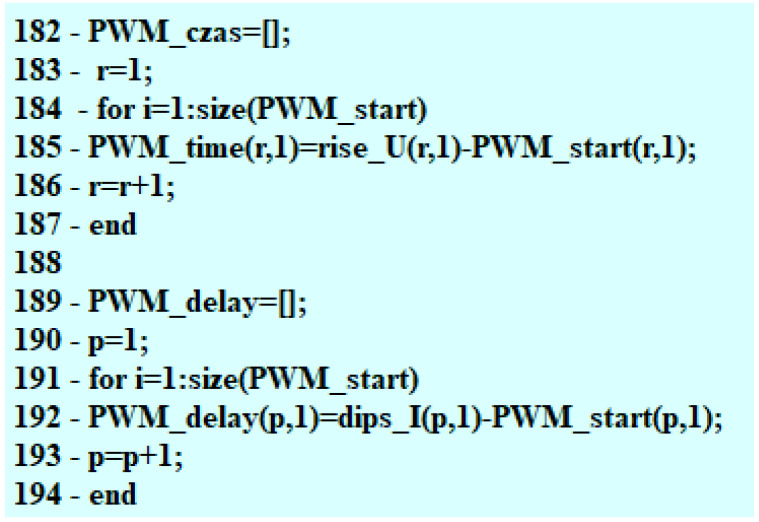
Assigning the time-ranges spotted by the algorithm.

The split-time matrix is created. The algorithm moves successively along the length of the matrix including the time of the voltage decrease as well as the time of voltage returning to its initial value. When the whole period is completed, the times spotted by the algorithm are assigned to the matrix. Due to this, the betweentime_to_endpeak matrix is created (Figure 6).

**Figure 7:**
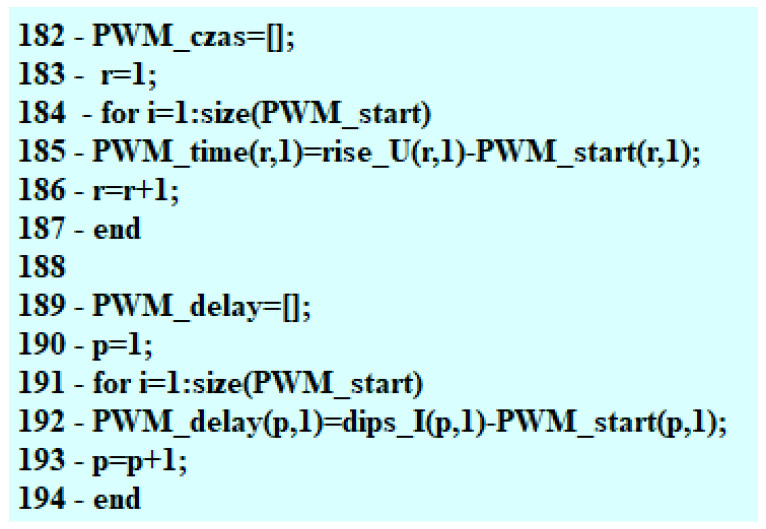
Finding the local minimum of the current intensity.

The algorithm seeks the local minimum and only takes into account the intervals, where the value of current intensity is different from zero (Figure 7). The islocalmin function is being defined. The sample, where the local minimum is located, gets recorded in the algorithm memory. The algorithm keeps verifying, whether there is only one minimum for each injection.

**Figure 8:**
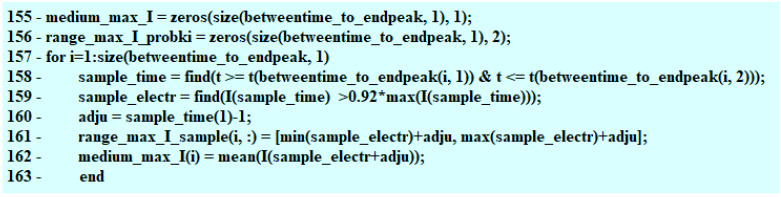
Final part of the search for the needle lifting phase.

Subsequently, the time reading at the point of the PWM signal beginning is compared to the time at the point of the needle lifting. On the basis of the difference between two times, the value of delay in the moment of starting the fuel flow is determined (Figure 8).

As feedback for the user, the program gives the value of the current intensity at the point of the opening of the fuel flow as well as the time that lapsed between the moment of the PWM signal beginning and opening of the fuel flow. These results are presented in the final table and can be applied to the analyzed characteristics, as shown in the calculation examples in the next section of the paper.

The algorithm finishes the iteration cycle with the flashing of six important quantities on the user panel. They are displayed in a sequence from the left side, as shown in Table 1:

- *PWM*—Pulse-Width Modulation [s];
- $t_{op}$—the delay time of the needle lifting point from the start PWM [s];
- *Start/End*—Steady-state start and end [s];
- $I_{max}$—value of electric current intensity in steady state (maximum) [A];
- $U_{L\;max}$—value of electric voltage at the maximum of induction peak [V] and value occurrence phase [s];
- $I_{op}$—electric current intensity at the point of needle lifting [A] and value occurrence phase [s].

**Table 1:** User panel filled with selected values.

PWM [s]	Delay in lifting the needle [s]	The time interval for achieving a constant electricity current value and the intensity value Start [s] - End [s] || Average value of the current intensity [A]	Maximum values of voltage peaks [V] for successive samples [V]	Electric current [A] at the point where the fuel flow is opened

The values can be displayed on an ongoing basis. Comparison of these values with the model of the executive element renders the values in the form of a difference, which is the evidence of faulty operation. After implementation of this method/algorithm in the controller’s memory, automatic control of the system operation will be obtained. This method may be applied in the control of various executive elements powered and controlled by the electric current.

## 6. Example Calculation Results

On the basis of the developed algorithm and computational program, analyses were carried out of the current waveforms of a fuel injector of a spark-ignition internal combustion engine marked as AR32104, equipped with injectors marked as 0 280 155 769. The use of different control parameters made it possible to conduct experiments to verify the operation of the injector under different conditions.

**Figure 9:**
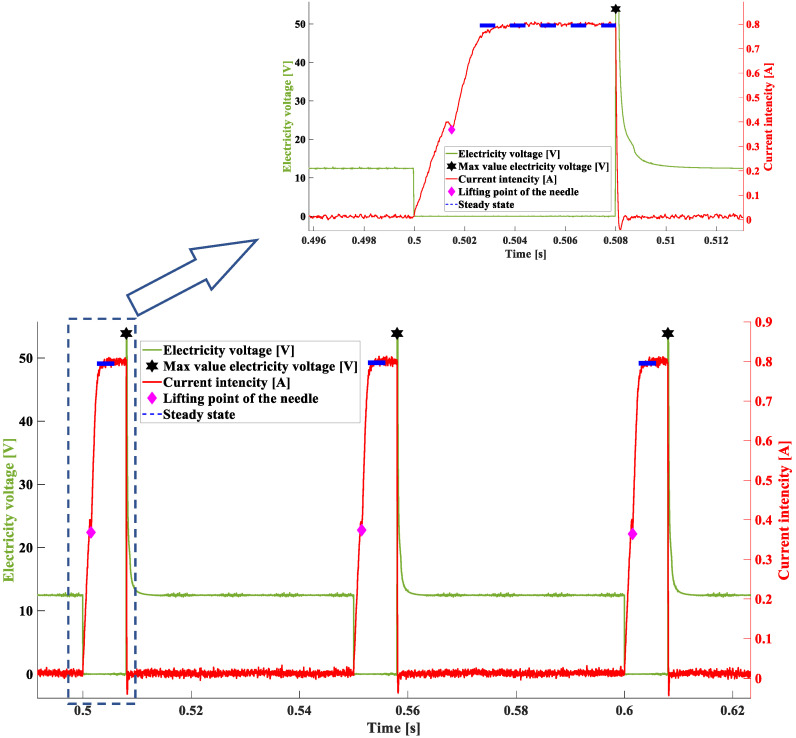
Characteristic values obtained in successive voltage–current waveforms representing the dosage of the fuel injector—exp. I—8 ms, 0.4 MPa.

In the first case, analyses were carried out for a time of 8 ms and a pressure of 0.4 MPa (exp. I), which corresponds to the operation of the injector at a low engine speed and high load. Figure 9 shows the result of the algorithm operation. In the successive voltage–current waveforms, based on the algorithm operation, the values that were sought have been marked:

- Dark blue dotted line—$I_{max}$—value of electric current intensity in steady state (maximum) [A];
- Black star—$U_{L\;max}$—value of electric voltage at the maximum of induction peak [V];
- Pink rhombus—$I_{op}$—electric current intensity at the point of needle lifting [A].

Figure 9 illustrates a single fuel injection described by the electric current quantities; the obtained values are marked. Green line represents the waveform of voltage in the injector coil. The maximum of the induction jump was determined within this waveform. When this value decreases, the short is indicated in the supply system or in the injector coil. The red line represents the waveform of the electric current. The characteristic twist in the waveform at the point where t = 0.5015 s indicates the needle lifting. Observation of this point enables determination, whether movement of the needle occurred, and the ordinate value can be related to the level of fuel pressure before the injector [8]. The level of the steady state of the electric current (dark blue, dotted line) indicates a change in resistance in the electrical system.

Table 2 shows the obtained results of the significant values that were measured and calculated for the tested injector. This study showed that the developed program unambiguously determined the sought-after characteristic points of the operation and calculated the relevant quantities for successive injector dosages.

**Figure 10:**
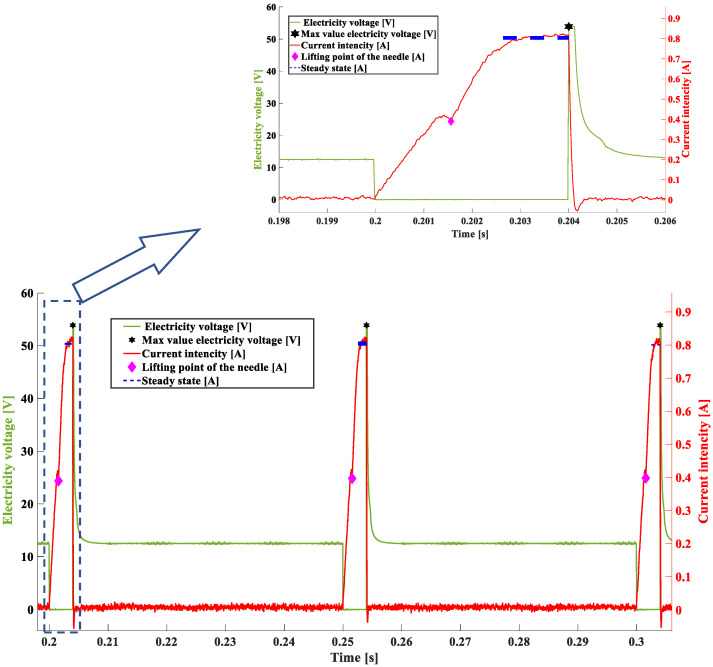
Characteristic values obtained in successive voltage–current waveforms representing the dosage of the fuel injector—exp. II—4 ms, 0.4 MPa.

In the next experiment, the characteristics recorded for a time of 4 ms and a pressure of 0.4 MPa were studied (exp.II). An injection time and pressure suitable for average engine loads were used. The values of the points found were as follows: the delay in lifting the needle (approx. 1.58 ms), the induction jump after the control pulse was switched off was approximately 54 V (at 12 V supply) (which indicating the efficiency of the electrical supply system), and the electrical current at the needle lift point was approximately 0.4 A (Figure 10, Table 3), which are the model values for this injector. The characteristic parameters confirm the operation of the injector without damage.

**Figure 11:**
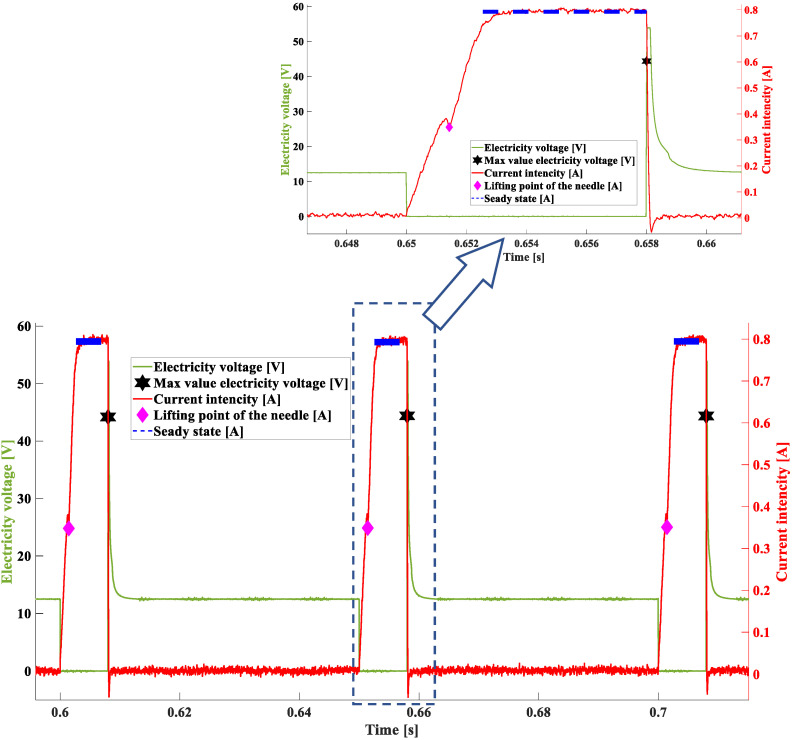
Characteristic values obtained in successive voltage–current waveforms representing the dosage of the fuel injector—exp. III—8 ms, 0.2 MPa.

In the successive experiment, the characteristics recorded for a time of 8 ms and a pressure of 0.2 MPa were studied (exp. III). The fuel pressure value was the lowest pressure value that was used in the control—engine idle. The time of 8 ms was a relatively “long” time. The quantities adopted in this experiment allow for confirmation of the search for the characteristic points of the injector operation (Figure 11 and Table 4) after the application of variables different from those used in previous studies. The values obtained in the user panel (Table 4) were the model data for this injector and the control parameters, showing no modification (induction jump of about 45 V and current at the needle lift point of about 0.35 A). Comparing the current at the needle lift point between Table 3 and Table 4, it can be seen that a reduction in the fuel pressure from 0.4 MPa to 0.2 MPa resulted in a reduction in the current value at this point from 0.4 A to 0.35 A, which is in line with the expectations [8].

## 7. Discussion

The monitoring of fuel injectors of internal combustion engines can be carried out on the basis of the observation of changes in the current value waveforms. As shown in the results from the studies presented in [33], the determination of the instantaneous state of the system is possible on the basis of the observation of the characteristic points of the current waveform and the additional manual determination of the characteristic values. This process is encumbered with a significant expenditure of time and prevents the detection of rapidly varying local changes in time signals.

The dependencies presented in the paper and the calculation algorithm made it possible to develop a program for automatically determining the relevant values of fuel injector current waveforms. Automatic detection of characteristic values related to the operation of the injector makes it possible to track its current operation, regardless of the engine’s instantaneous control values. The program allows for the observation of rapidly changing signal values in successive duty cycles and also allows for the search for possible discrepancies, which may be a symptom of a change in the technical condition of the injector or the injection system. Presentation of the results in the form of drawings and tables makes it much easier to analyze the characteristic points and compare the determined values with the model values for a specific injection system. An additional important feature of the program is the presentation of test results from a number of consecutive injector cycles, which makes it possible to later compare the obtained values. The essence of the method is that it can be implemented in the internal combustion engine control unit (ECU). Automatic verification of changes occurring in the current waveforms will enable the feedback response of the control system as a protective response already in the initial states of change in the technical condition or damage to the actuator.

The developed program for the automatic detection and determination of the characteristics of the current signals of fuel injectors significantly expands the possibilities of the monitoring of these elements carried out thus far. It is also becoming a tool, the use of which can enable ongoing adjustments to the settings of individual components of the engine’s injection system, for example, to meet the emission standards for toxic exhaust components.

The sample tests of the fuel injector presented in this paper, using the developed program, made it possible to determine its characteristic values depending on the engine speed and load. The results show that regardless of the engine speed—times of 4 and 8 ms, and for different loads—pressures of 0.2 and 0.4 MPa—the program determined the sought-after characteristic values. Analyzing the results presented in Table 2 and Table 3—pressure of 0.4 MPa during the tests—and Table 4—pressure of 0.2 MPa—it can be seen that the change in fuel pressure results in a decrease in the current intensity at the point of needle lifting (change from a value of >0.36 A to a value of <0.35 A). This is consistent with earlier studies that showed that the intensity of the electric current depends directly on the fuel pressure. The higher the fuel pressure, the higher the current intensity during the needle lifting phase. These differences are consistent with the results of the authors’ earlier studies. Additionally related to this parameter is the delay time of the needle response, which is shorter at lower fuel pressure (Table 4)—a reduction from a value of 0.00156 s to a value of 0.0014 s. Confirmation of the effectiveness of the method as well as the correctness of the search for characteristic values are the results of tests calculated from signals recorded in the experiments where the time was 8 ms and 4 ms, and the fuel pressure had a constant value of 0.4 MPa. The results of these studies, presented in Table 2 and Table 3, have significant reproducibility. The presented sample results came from tests of the automatic software operation that were performed using fully operational fuel injectors. If any damage occurred, the changes would be very pronounced.

## 8. Conclusions

The performed experiments and the presented sample results show that the developed computational program created on the basis of the adopted assumptions of the computational algorithm makes it possible to detect important features contained in the current–voltage characteristics of the fuel injector during its normal operation. The program identifies characteristic locations on the recorded current curves and automatically calculates the values sought by marking their position on the characteristic curves and posting the results in a table.

The code fragments presented in Section 5 allowed for the correct detection of characteristic points within the voltage–current waveform, which is discussed in Section 6. Observation of these points allows for fast verification (diagnostics) of the fuel injector operation. This method may be implemented directly in the memory of the controller (ECU), and after the modification of its design (addition of the assembly measuring the current intensity), the diagnostics may be performed on an ongoing basis without using additional diagnostic modules. The information on the occurrence of failures may be passed on directly to the vehicle user by means of messages from the on-board computer. At the same time, after the additional algorithms in the ECU have been used, the controller can undertake action aimed at improving the combustion process in the engine or effectively pressure toward the visit in the car service/garage.

What is important, is that this method allows for the early detection of failures before there is any secondary damage to the engine’s power management system and environmental pollution due to improper engine operation, or before the damage is detected by the on-board diagnostics system.

Bearing in mind the foregoing, further research should be conducted directly on the internal combustion engine control unit to validate the developed algorithm for analyzing the technical condition of a specific sensor during normal operation.

## Figures and Tables

**Table 2:** User panel filled with selected values—exp. I—8 ms, 0.4 MPa.

	PWM [s]	Delay in lifting the needle [s]	The time interval for achieving a constant electricity current value and the intensity value Start [s] - End [s] || Average value of the current intensity [A]	Maximum values of voltage peaks [V] for successive samples [V]	Electric current [A] at the point where the fuel flow is opened
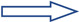	0.008 0.00802 0.00802 0.00802 0.00802 0.00802 0.00802 0.00802 0.00802 0.00802 0.00802 0.00802 0.00802	0.00148 0.0015 0.0015 0.0015 0.0015 0.00154 0.00152 0.0015 0.0015 0.00148 0.0015 0.00148 0.0015	0.00258 - 0.008 || 0.79389 0.0526 - 0.058 || 0.79554 0.10266 - 0.108 || 0.79497 0.15266 - 0.158 || 0.79572 0.20264 - 0.208 || 0.79462 0.2526 - 0.258 || 0.79531 0.30258 - 0.308 || 0.79415 0.35262 - 0.358 || 0.79614 0.40264 - 0.408 || 0.79414 0.45258 - 0.458 || 0.79529 0.5026 - 0.508 || 0.7945 0.55262 - 0.558 || 0.79674 0.60262 - 0.608 || 0.79531	"53.885 for t=0.008" "53.885 for t=0.058" "53.885 for t=0.108" "53.885 for t=0.158" "53.885 for t=0.208" "53.885 for t=0.258" "53.885 for t=0.308" "53.885 for t=0.358" "53.885 for t=0.408" "53.885 for t=0.458" "53.885 for t=0.508" "53.885 for t=0.558" "53.885 for t=0.608"	"0.36435 for t=0.00148" "0.37111 for t=0.05148" "0.36918 for t=0.10148" "0.36532 for t=0.15148" "0.37015 for t=0.20148" "0.37691 for t=0.25152" "0.36532 for t= 0.3015" "0.37401 for t=0.35148" "0.36242 for t=0.40148" "0.36918 for t=0.45146" "0.36822 for t=0.50148" "0.37401 for t=0.55146" "0.36435 for t=0.60148"

**Table 3:** User panel filled with selected values—exp. II—4 ms, 0.4 MPa.

	PWM [s]	Delay in lifting the needle [s]	The time interval for achieving a constant electricity current valueand the intensity value Start [s] - End [s] || Average value of the current intensity [A]	Maximum values of voltage peaks [V] for successive samples [V]	Electric current [A] at the point where the fuel flow is opened
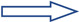	0.004 0.00402 0.00402 0.00402 0.00402 0.00402 0.00402	0.00156 0.00158 0.00154 0.00158 0.00158 0.00158 0.00158	0.00256 - 0.004 || 0.80115 0.05258 - 0.054 || 0.80561 0.10252 - 0.104 || 0.80095 0.15258 - 0.154 || 0.80691 0.20264 - 0.204 || 0.80354 0.25258 - 0.254 || 0.80426 0.3025 - 0.304 || 0.80047	"53.885 for t=0.004" "53.885 for t=0.054" "53.885 for t=0.104" "53.885 for t=0.154" "53.885 for t=0.204" "53.885 for t=0.254" "53.885 for t=0.304"	"0.40301 for t=0.00156" "0.39238 for t=0.05156" "0.39431 for t=0.10152" "0.38754 for t=0.15156" "0.38947 for t=0.20156" "0.39721 for t=0.25156" "0.39817 for t=0.30156"

**Table 4:** User panel filled with selected values—exp. III—8 ms, 0.2 MPa.

	PWM [s]	Delay in lifting the needle [s]	The time interval for achieving a constant electricity current value and the intensity value Start [s] - End [s] || Average value of the current intensity [A]	Maximum values of voltage peaks [V] for successive samples [V]	Electric current [A] at the point where the fuel flow is opened
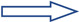	0.008 0.008 0.008 0.008 0.008 0.008 0.008 0.008 0.008 0.008 0.008 0.008 0.008 0.008 0.008	0.00144 0.00142 0.00142 0.00142 0.00142 0.00142 0.00144 0.00142 0.00142 0.00142 0.00144 0.00142 0.00144 0.00142 0.00144	0.00272 - 0.008 || 0.79558 0.05254 - 0.058 || 0.79255 0.10258 - 0.108 || 0.79449 0.1526 - 0.158 || 0.79299 0.20268 - 0.208 || 0.79562 0.25262 - 0.258 || 0.79331 0.30254 - 0.308 || 0.79466 0.35256 - 0.358 || 0.79319 0.40264 - 0.408 || 0.79441 0.45256 - 0.458 || 0.79243 0.5026 - 0.508 || 0.79415 0.55258 - 0.558 || 0.79267 0.60264 - 0.608 || 0.79428 0.65254 - 0.658 || 0.79298 0.7026 - 0.708 || 0.79471	"44.4667 for t=0.008" "44.3927 for t=0.058" "44.4042 for t=0.108" "44.5291 for t=0.158" "44.5209 for t=0.208" "44.35 for t=0.258" "44.304 for t=0.308" "44.4847 for t=0.358" "44.378 for t=0.408" "44.4207 for t=0.458" "44.5307 for t=0.508" "44.3517 for t=0.558" "44.189 for t=0.608" "44.3878 for t=0.658" "44.3599 for t=0.708"	"0.35372 for t=0.00144" "0.35372 for t=0.05142" "0.34599 for t=0.10142" "0.35179 for t=0.15142" "0.35469 for t=0.20142" "0.34889 for t=0.25142" "0.34986 for t=0.30144" "0.35275 for t=0.35142" "0.34792 for t=0.40142" "0.34405 for t=0.45142" "0.34986 for t=0.50144" "0.35469 for t=0.55142" "0.34792 for t=0.60144" "0.34889 for t=0.65142" "0.35082 for t=0.70144"

## Data Availability

Not applicable.
